# Optimization of CT calcium scoring doses on the General Electric Discovery single-photon emission computed tomography/CT D670, 8-slice scanner

**DOI:** 10.1259/bjrcr.20160023

**Published:** 2016-11-02

**Authors:** Clare Jacobs

**Affiliations:** Department of Medical Physics and Clinical Engineering, Nottingham University Hospitals Trust, Nottingham, UK

## Abstract

Manufacturer-recommended exposure mA was typically resulting in 3–5 times greater patient doses for calcium score scans compared with other dedicated CT scanners at Nottingham University Hospitals. Image noise was used as a measure of image quality in phantom and patient data. The noise was quantified from the standard deviation in Hounsfield units within regions of interest in the myocardium. Noise in phantom data was found to vary linearly with the inverse square root of the applied mAs. It was assumed that a linear relationship would also apply to patient data but it was predicted that the linear gradient would vary between patients owing to differing patient size and composition. This noise model was used to calculate the exposure mA required to achieve a target noise level of 25 Hounsfield units in the myocardium for each patient. To maintain the image quality for patients of different sizes, three measures of size, weight, body mass index (BMI) and lateral dimension, were all tested for goodness of fit to the noise model. It was found that BMI correlated best with the noise model for small patients, and therefore, BMI was chosen as a measure of patient size for the revised mA table. Using this methodology, doses to small patients were reduced by a factor of four compared with manufacturer-recommended settings.

## Background

The National Institute for Health and Care Excellence (NICE) guidelines for patients with chest pain of recent onset was published in 2010. Its aim was to set a pathway for diagnosing patients who have angina-like symptoms but no history of cardiac disease.^1^ The guidelines recommend performing CT calcium scoring as a screening test for patients at low risk of coronary artery disease, as the presence of coronary calcification is a known marker for atherosclerosis.^2^ In principle, calcium scoring is an imaging technique that uses gated CT to acquire pictures of the coronary vessels during phases of least cardiac motion. A threshold of 130 Hounsfield units (HU) is used for defining calcified regions.^3^ The Agatston score is a method of quantifying the degree of calcification whereby the area of the lesion is multiplied by a weighting factor (determined by the CT number) and the scores of all the affected slices are summed.^4^ The NICE guidelines require classification of patients into three groups; those with a score of 0, who do not need any further tests; those with a score between 1 and 400, who will need to have a CT coronary angiography; and those with a score > 400, who will go on to have conventional angiography. The upper threshold of 400 has been shown to correspond to patients with advanced plaque disease, with 90% specificity for at least one obstructive coronary lesion.^5^ A score of zero has a high negative-predictive value (95%) for excluding significant coronary artery disease.^3^

## Purpose of study

Owing to an increase in demand for CT coronary angiography and other services in the main CT department of the Nottingham University Hospitals, the possibility of performing calcium scoring on the Discovery NM/CT 670 scanner (GE Healthcare, Waukesha, WI) in the nuclear medicine department was explored. This is a gamma camera with an 8-slice CT that has the functionality to acquire gated CT. This scanner does not have the capability to perform dose modulation when acquiring gated CT; therefore, manual exposure parameters must be used. The initial acquisition parameters were set to those recommended by the manufacturer, which are documented in their Smartscore user manual.^6^ The first four patients scanned on the Discovery scanner showed good image quality but high radiation doses compared with other dedicated CT scanners in the hospital; dose–length product range 150–250 mGy cm compared with 30–50 mGy cm. Therefore, a calcium scoring service using the Discovery scanner could only be justifiable if the radiation doses could be brought down to a similar level. The purpose of this report is to describe the methodology that was used to achieve this.

## Aim

Image quality can be assessed in many ways, both qualitatively and quantitatively. The aim of this study was to look at image noise in the first instance and try and quantify this. Later on in the report, other factors affecting image quality, including temporal resolution and patient motion, are also described.

The primary method of dose optimization was to use a CT thorax phantom to explore the noise characteristics of the scanner and derive a methodology for adjusting the mA to achieve a target noise level.^7^ The applicability of this methodology to patient data was then tested. Exposure settings must be varied with patient size to maintain image quality; therefore, different measures of patient size [lateral dimension, weight and body mass index (BMI)] were tested for correlation with the noise model. This builds on the study by McCollough et al^8^ who used the thorax phantom with additional attenuation rings to model small, medium and large patients. The purpose of their study design was to optimize the exposure parameters for each size of phantom to maintain image noise and then use these settings for patient exposures.

## Method

**Figure 1.**
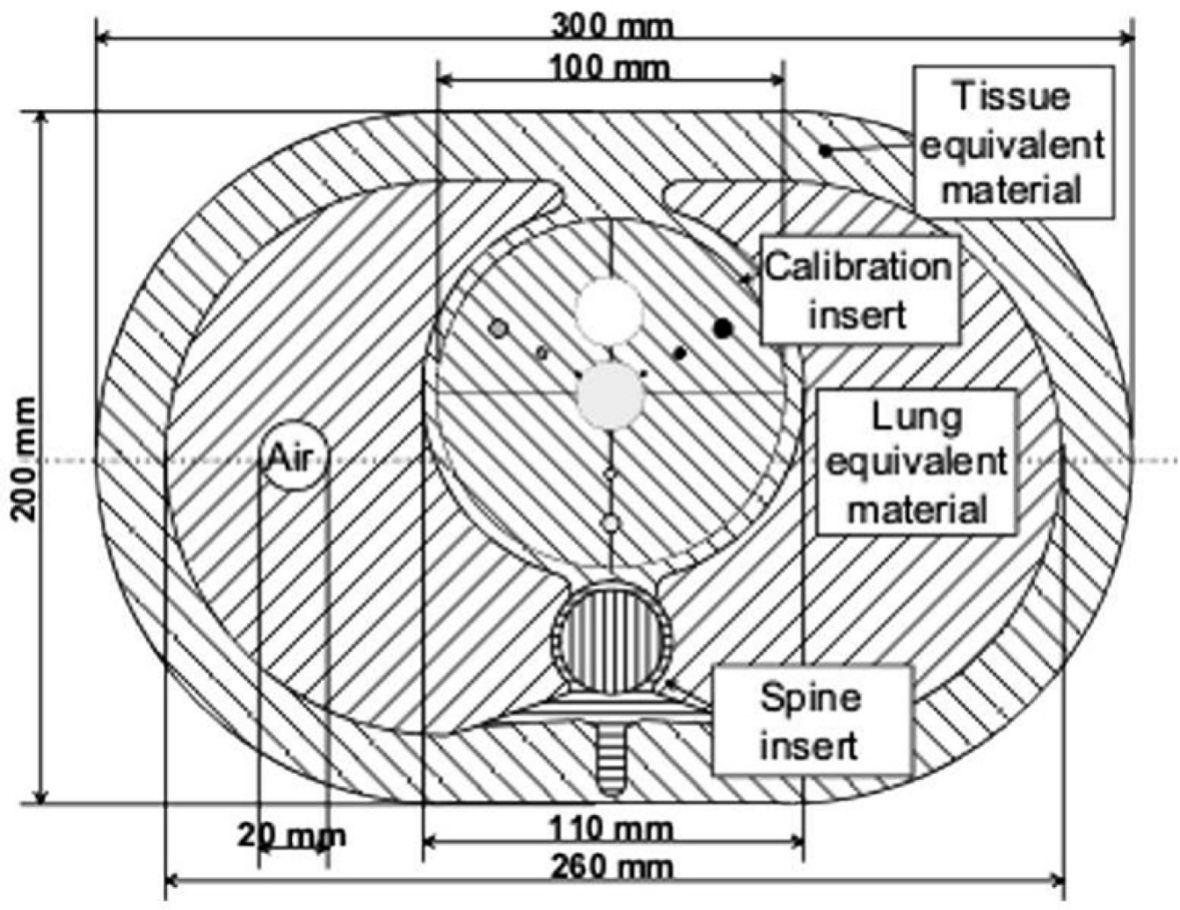
Cross-sectional view of the calcium-scoring phantom. The view consists of lung, heart and spinal regions.

The calcium-scoring phantom (QRM GmbH, Moehrendorf, Germany) models the CT attenuation properties of organs in the thorax (Figure 1). This phantom was used to investigate how image noise varies with tube current time product (mAs) to obtain a characteristic noise plot for the Discovery scanner and also the Siemens Definition AS (Siemens, Erlangen, Germany) stand-alone CT scanner. The comparison of noise characteristics would allow identification of inherent differences in the performance of the two scanners.

For valid comparison between the scanners, the acquisition and reconstruction protocols must be similar. Images were acquired in the axial mode using a tube voltage of 120 kVp. Because image noise differs across the field of view (FOV), it was ensured that the phantom was positioned centrally within the bore of the scanner.^3^ The X-ray tube rotation time for calcium score acquisitions is scanner specific and unalterable; therefore, the mA was the only parameter that was varied between repeat acquisitions.

The thickness and number of slices that each scanner can acquire per rotation is different; the Discovery is an 8-slice scanner with a slice thickness of 2.5 mm and the Definition AS is a 32-slice scanner with a slice thickness of 1.2 mm. It was ensured for the comparison that both datasets were reconstructed as 5 mm slices, as this was the smallest slice thickness common to both scanners. Images were reconstructed using filtered back projection (FBP), as this was the only reconstruction method available on the Discovery scanner.

**Figure 2.**
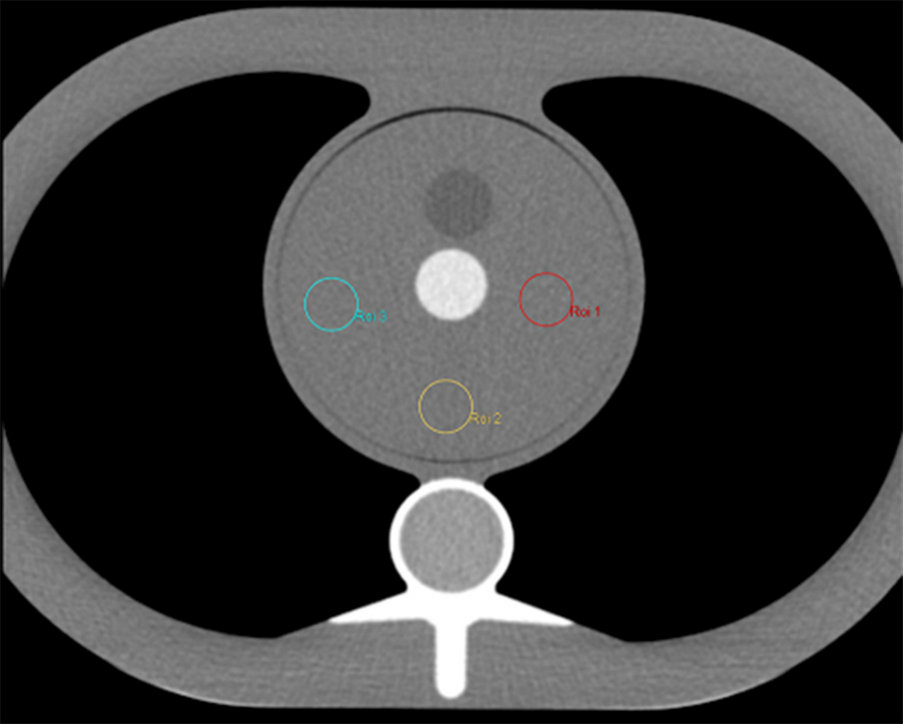
A single reconstructed slice through the CT phantom. Three circular regions of interest are drawn in the myocardial equivalent region to assess image noise.

**Figure 3.**
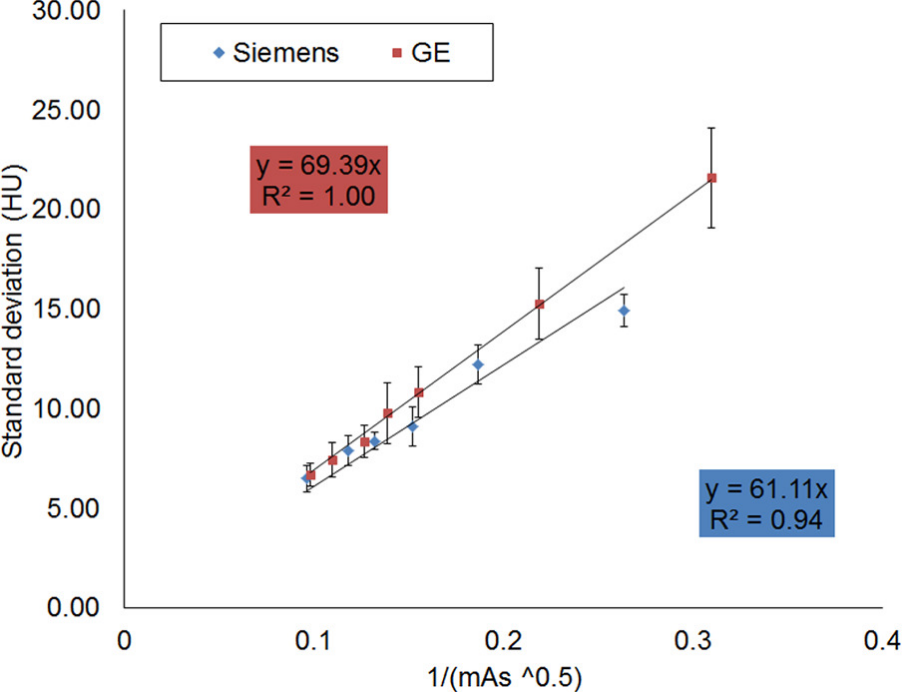
Characteristic noise plot for both the Siemens Definition AS (Siemens, Erlangen, Germany) and the GE Discovery (GE Healthcare, Waukesha, WI) scanners. Error bars correspond to the variation in noise (± 1 standard deviation) of Hounsfield units (HU) in the three regions of interest.

The datasets were loaded into the Hybrid viewer program on a Hermes workstation (Hermes Medical Solutions, Stockholm, Sweden). Three circular regions of interest (ROIs) of 2 cm diameter were created in the central “heart” region in one of the datasets on one slice. These ROIs were copied to the same slice and the same locations for all the other datasets from that scanner (Figure 2). An average of the standard deviation of HU in these three ROIs was used to determine the average noise. Error in measured noise was determined from the standard deviation of measured noise in the three ROIs (Figure 3).

For a filtered back projection (FBP) reconstruction of the CT data, it is known that the image noise will vary with the inverse square root of the number of photons per pixel.^9^ The number of photons is proportional to the tube current, therefore the noise should vary inversely with the square root of the tube current time product. The standard deviation of pixel values in HU is a measure of the noise in the image.^8^ A plot of the noise characteristics for the two scanners is shown in Figure 3.

It can be seen in Figure 3 that there is a linear relationship between the measured noise and inverse square root of the mAs for both scanners. Despite differences in hardware (X-ray tube, detector composition/size), and the reconstruction smoothing kernel, the overall noise performance of both scanners is similar, with the gradient of the linear fit differing by approximately 12%. The equations of best fit have been forced to pass though the intercept since it is intuitive that, as the applied tube current increases, the noise gets smaller, tending to zero. This phantom comparison shows that if the images from the Definition AS scanner are deemed diagnostic for a lower patient dose, then the doses could also be lowered for the Discovery scanner. The next stage was to determine the methodology for achieving the dose reduction. Under normal use, the Definition AS scanner uses automatic mA modulation for calcium scoring, so it is not possible to directly copy the exposure parameters to the Discovery scanner.

## Patient images

To translate what has been observed with the phantom data to patient data, it was assumed that a linear relationship would also result if a patient were imaged repeatedly at different values of mAs. It would be expected that the gradient of the characteristic noise plot would vary between patients of different size and composition, but that the noise would tend to zero as the mAs increased. To determine the linear equation for each patient, a minimum of two points on the line are needed. It is possible to derive this because the fit can be forced to pass through the intercept. The second point on the line was the measured noise at the known exposure mAs. The gradient of this linear fit was then used to calculate the mA required to achieve the target noise level, given that the tube exposure time per rotation is fixed at 0.52 s.

## What image noise levels should we aim for?

Looking at a number of clinical calcium score studies obtained on the Definition AS scanner, the noise levels ranged between 25 and 28 HU. For the initial four patients scanned on the GE Discovery scanner, the noise levels ranged from 10 to 21 HU (Table 1). The typical noise level of electron beam data, which was once the gold standard for calcium scoring acquisitions, has been documented as 24 HU.^8^ Therefore, it was decided that 25 HU would be a reasonable starting point in the optimization process.

## Patient population

Calcium scoring assessment was made on 25 patients who had been referred from the rapid access chest pain clinic at Nottingham City Hospital over an 8-month period. The referral population consisted of 19 females and 6 males who had a NICE risk score between 10% and 30%. Patients with arrhythmias were excluded from the test. No patients were specifically prescribed rate controlling medication prior to their appointment, although some patients were already on beta blocker medication.

## Methodology for patient imaging

It was very important to minimize patient motion during imaging; therefore, a hyperventilation technique was used and the images were acquired during the end-inspiratory breath-hold period. For optimal image quality, the patients were centred in the scanner using laser lights. Depending on the scanner hardware specifications, a patient’s heart rate can also be an issue. It was recommended for the Discovery scanner that the heart rate should be < 75 beats per minute (bpm); therefore, not all patients who were referred could be scanned. On both scanners, images were acquired using prospective gating to minimize patient dose.

The image analysis was performed in much the same way as for the phantom data. A 2 cm circular ROI was drawn on four different CT slices within the myocardium for each patient and the standard deviation in HU were recorded. The mean of these measurements was used to determine the average noise. Using the linear fit model, the gradient of the fit was computed for each patient and used to calculate the mA required to produce image noise of 25 HU in the myocardium. The calculated mA to achieve a noise index of 25 HU in the first four patients who were scanned is given in Table 1.

**Table 1.** The table lists the actual mA used and the computed mA required to obtain an image with a target noise level of 25 HU for the first four patients scanned on the Discovery scanner

Patient number	Mean noise level (standard deviation HU)	mA used	Calculated gradient	Calculated mA required to achieve a noise level of 25 HU	Original dose (mSv)	Modified dose (mSv)
Patient 1	18.6	200	263	111	2.1	1.2
Patient 2	16.8	250	266	113	3.4	1.5
Patient 3	21.5	300	373	222	3.4	2.5
Patient 4	10.6	250	168	45	2.5	0.4

DLP, dose-length product; HU, Hounsfield units. The conversion from DLP (mGycm) to effective dose (mSv) was achieved by multiplying the DLP by the factor 0.014, listed in table A.2. of ICRP publication 102.^9^ Discovery scanner manufactured by GE Healthcare, Waukesha, WI.

Results in Table 1 show that using the linear model for noise, the doses for Patient 4 could be lowered by a factor of six and approximately halved for Patients 1 and 2.

## Scaling mA for patients of different sizes

**Figure 4.**
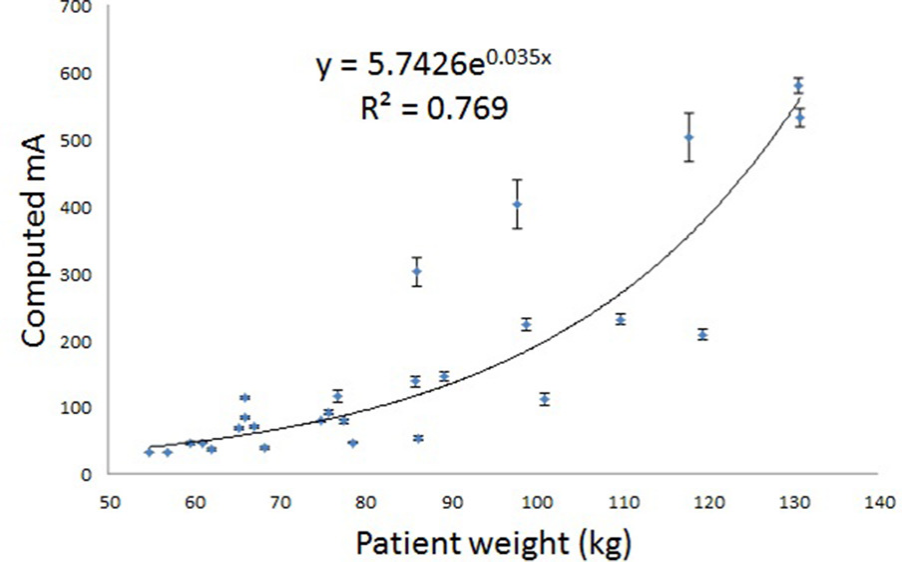
Plot of computed mA *vs* patient weight (kg), to give image noise of 25 HU in the myocardium for the first 25 patients scanned using the Discovery (GE Healthcare, Waukesha, WI) scanner. The error bars denote the error in calculating the mA from the variation of noise measured in the regions of interest.

**Figure 5.**
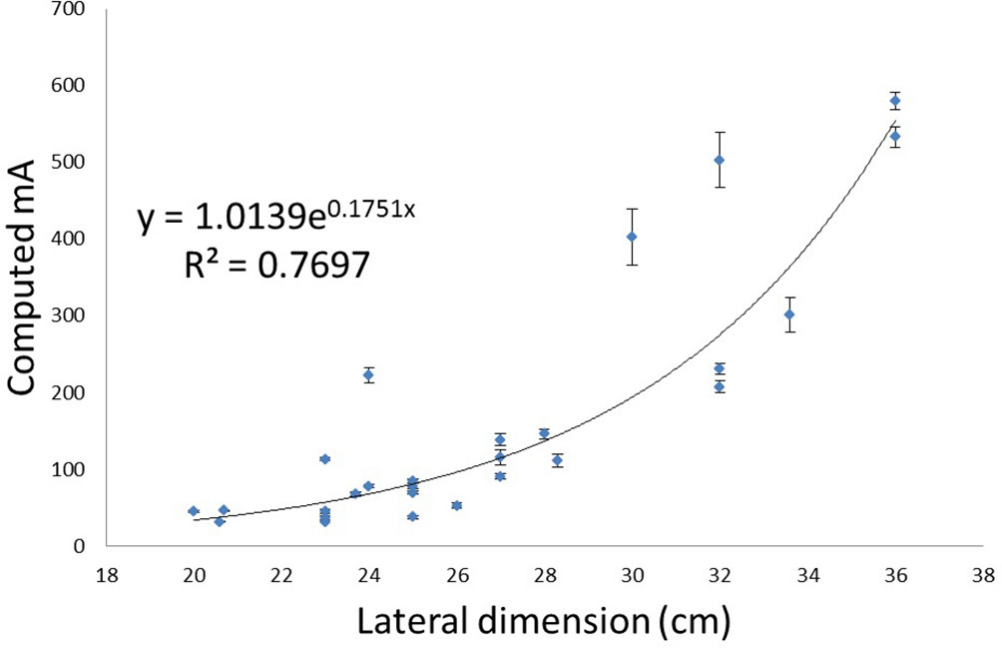
Plot of computed mA *vs* lateral dimension (cm), to give an image noise of 25 HU in the myocardium for the first 25 patients scanned using the Discovery (GE Healthcare, Waukesha, WI) scanner.

**Figure 6.**
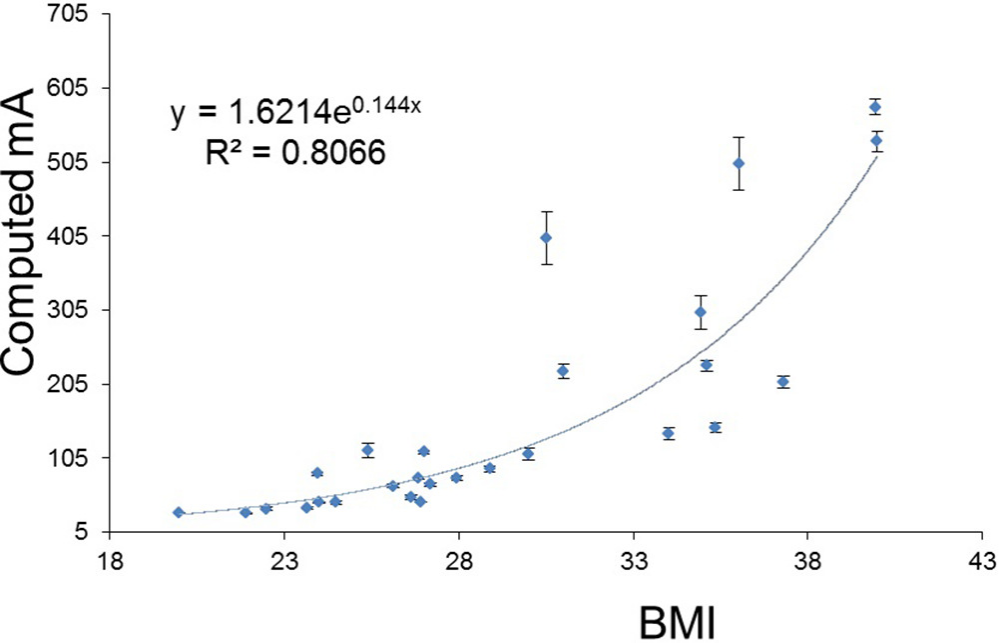
Plot of computed mA *vs* patient body mass index (BMI), to give an image noise of 25 HU in the myocardium for the first 25 patients scanned using the Discovery (GE Healthcare, Waukesha, WI) scanner. BMI (Body mass index)

As patients have different attenuating properties owing to size and composition, the mA will need to be varied for patients of different sizes to obtain the same target noise level of 25 HU.^9^ There is discussion in the literature as to how best to categorize patients; the commonly suggested criteria include weight, BMI or lateral dimension. In a study by McCollough et al,^8^ they found that the lateral patient dimension correlated best with noise. The aim of this study, therefore, was to test which of these measures of patient size best fitted the noise model for our patient population. The “computed mA” was plotted against weight, lateral width (as measured from the lateral scout of the patient) and BMI (Figures 4–6). An exponential best fit was used in each case and the least squares fitting parameter was used to determine the closeness of the fit to the real data. The error in the computed mA was calculated from the percentage variation of noise measured in the ROIs (± 1 standard deviation).

Figures 4–6 show a similar pattern of results, with smaller patients fitting better with the noise model than larger patients. BMI was found to correlate best with the noise model for those with a BMI <31, therefore, this relationship was used to set a new manual exposure mA table (Table 2). For ease of use, the patients were classified into four groups; small, medium, large and very large. The manufacturer-recommended exposure mA is listed in the last column for comparison. It can be seen from the table that the new mA settings for the small patients are a factor of four lower than that recommended by the manufacturer.

**Table 2.** mA table for performing calcium scoring on the Discovery D670 scanner based on patient BMI

Patient size	BMI	mA revised	GE-recommended mA
Small	24	45	200
Medium	27	110	230
Large	31	160	250
Very large	35	210	

BMI, body mass index. The revised settings (middle column) can be compared to manufacturer-recommended settings (last column) listed in the Smartscore user manual.^6^

**Figure 7.**
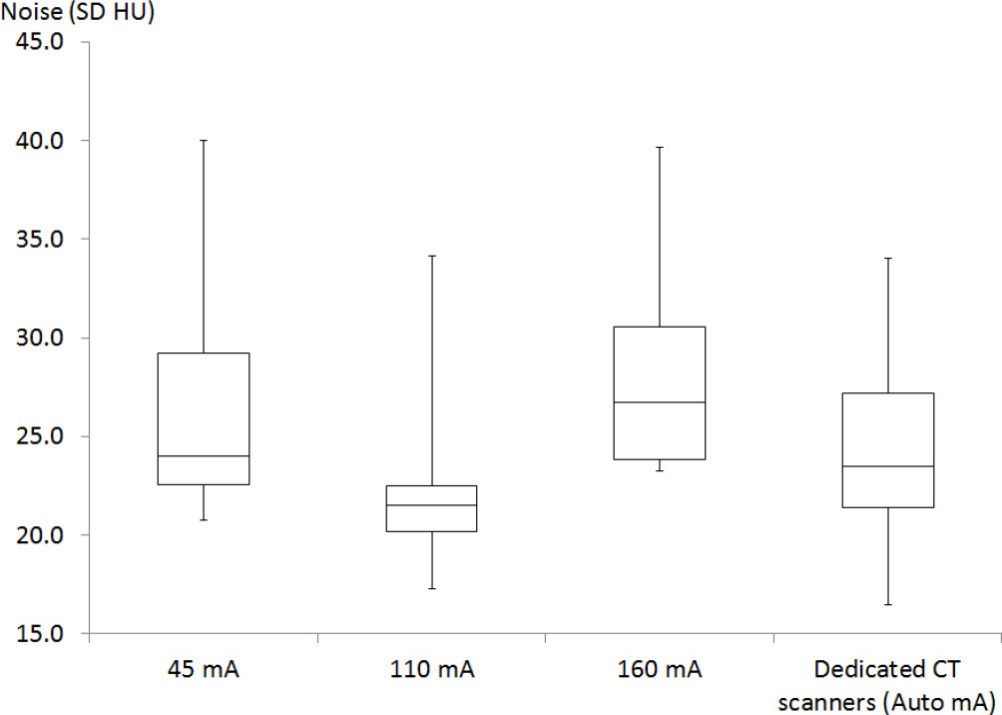
Box and whisker plot of the variation in noise measured for patients scanned using the mA settings in Table 2. Displayed are the maximum, minimum, median and interquartile range. For comparison, a plot of noise variation for 22 patients scanned on other dedicated CT scanners, where Auto mA is used, is shown on the right. HU, Hounsfield units; SD, standard deviation.

A total of 24 patients were scanned on the Discovery scanner using the revised mA table. The resulting image noise distributions are displayed as box and whisker plots for each mA setting in Figure 7. For comparison, the noise measured in a sample of 22 calcium score images obtained on other dedicated CT scanners in the hospital are also shown.

**Table 3.** A summary of the variation of noise (SD HU) measured in patient images after practical implementation of Table 2 for small (45 mA), medium (110 mA) and large patients (160 mA), compared with images obtained on other dedicated CT scanners

Noise (SD HU)	45 mA	110 mA	160 mA	Dedicated CT scanners (Auto mA)
Minimum	21	17	23	16
Lower quartile	23	20	24	21
Median	24	21	27	23
Upper quartile	29	23	31	27
Maximum	40	34	40	34
Mean	26	23	28	24

HU, Hounsfield units; SD, standard deviation.

Although only a small number of patients were scanned in each category, 10 patients at 45 mA, 6 at 110mA and 8 at 160 mA, overall there was good agreement with the target of 25 HU. For the “small” patient category, 7 of 10 patients achieved image noise ≤ 25 HU. The maximum noise measured in the small category was 40 HU for one patient. If the exponential fit equation in Figure 6 had been used for scaling mA with BMI rather than the 45 mA in Table 2, then 60 mA would have been used for this patient and thus the noise would have been lower. For the “large” patient group scanned using 160 mA, only three out of eight patients achieved the target of 25 HU or better, which indicates that the mA may need to be increased for this group. Upon comparison with the distribution seen for the dedicated CT scanners, the mean, median and range are similar to values of the small and medium-sized patients measured on the Discovery scanner (Table 3 and Figure 7).

## Discussion

The aim of this study has been to lower the patient doses for performing calcium scoring on the GE Discovery D670 scanner. This study has made an assumption that the linear noise relationship seen with phantom data when noise is plotted against the inverse square root of the mAs would also be applicable to the patient data. It has been shown in Figures 4–6 that this noise model is reasonable for the smaller patient population. BMI was found to correlate best with the noise model for those with BMI ≤ 31, and therefore BMI was chosen as a measure of patient size for computing the reference mA table. With the revised mA settings shown in Table 2, small patients with BMI ≤ 24 now receive one-quarter of the dose compared with the original manufacturer settings. Practical implementation of the revised exposure settings has shown that the mA will need to be increased for the large patient category to achieve the target noise of 25 HU (Figure 7 and Table 3). Future studies will involve refinements to the exposure settings in Table 2.

The work described in this study has focused on quantification of image noise as the measure of image quality. The quantification of image noise was simplified by the use of FBP reconstruction, which allowed the standard deviation of HU in ROIs drawn within the myocardium to be used as a measure of noise. These results cannot be used for direct comparison with iterative reconstructions of the same data because noise levels for iterative reconstructions follow a non-linear relationship.^10^

Unlike most assessments of image quality, where features in the whole cross-section of a patient need to be assessed, the specific nature of the application of CT for calcium scoring also helped simplify the optimization process. It is known that the radiation flux homogeneity, and hence noise, in the central region of the CT FOV differs from that at the edges, reportedly by as much as 50%.^11^ Fortunately, in the case of calcium scoring studies, the heart should usually be positioned close to the centre of the FOV; which minimizes additional noise variations due to flux field inhomogeneity.

## Patient motion

Image noise is not the only factor determining image quality for calcium scoring scans, motion due to patient breathing, arrhythmias and heart rate also have a significant impact. Scanner hardware specifications and reconstruction software will limit what can be reasonably achieved clinically and differ significantly between the two scanners described in this work.

**Figure 8.**
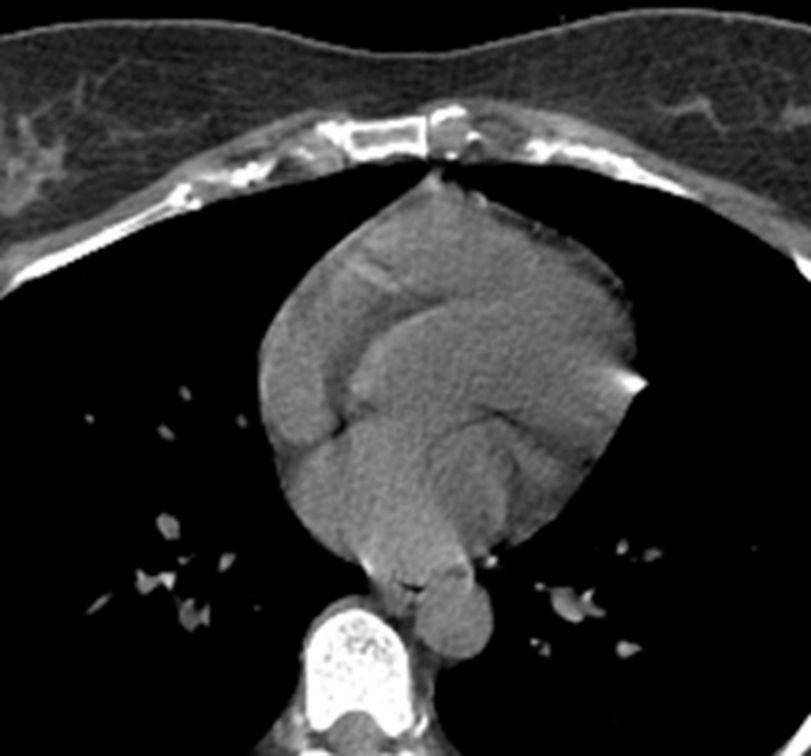
Calcium score images of a patient with a heart rate of 100 beats per minute. A high- density region above 130 HU is seen at the edge of the myocardium.

The time resolution of the CT scanner limits the maximum heart rates that can be imaged without suffering from significant motion artefacts due to cardiac movement. Figure 8 illustrates the effect of scanning a patient with a heart rate of 100 bpm on the Discovery scanner. There appears to be a large high density region at the pericardial border of the myocardium. The shape is not typical of coronary artery calcification and does not conform to a principal cardiac vessel. It would be most unusual to find a calcification this large in a small peripheral vessel.

**Figure 9.**
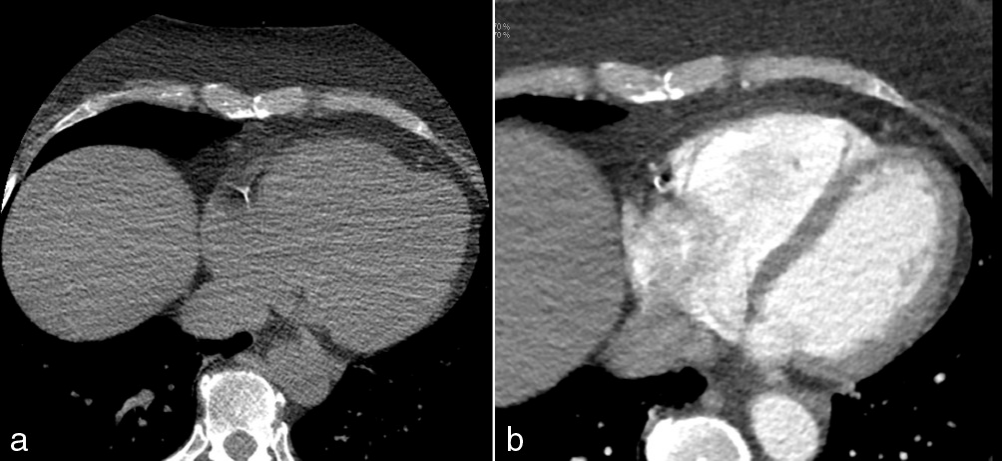
(**a**) Calcium score image of a patient acquired on the Discovery (GE Healthcare, Waukesha, WI) scanner with a heart rate of 65 beats per minute. Calcification is seen in the right coronary artery, which appears as a tail-shaped artefact. (**b**) Coronary angiography image of the same patient acquired on the Siemens Definition AS (Siemens, Erlangen, Germany) scanner without the tail-shaped artefact.

Time resolution is primarily determined by the X-ray tube rotation time. The minimum amount of data required to reconstruct a CT image for calcium scoring is 180º + the angle of the X-ray beam; this takes 0.238 s for the Definition AS scanner and 0.522 s for the Discovery scanner.^12,13^ The time resolution of the GE scanner is therefore approximately one-half that of the Definition AS scanner. The effect of the differing time resolutions is shown in Figure 9, where the same patient was scanned on both the Discovery and the Definition AS scanners for the calcium score and coronary angiography, respectively. For both scans, the patient’s heart rate was approximately 65 bpm. It can be seen in Figure 9a that the calcification in the right coronary artery territory has a tail-shaped artefact, which is not present in Figure 9b.

Motion due to breathing can also be a problem. This is more likely for patients scanned on the Discovery scanner, as the scan typically takes 20 s compared with 5 s on the Definition AS scanner. The different durations are due to the X-ray tube rotation time and slice coverage per bed position. Caution must therefore be applied when interpreting images that have been compromised by motion.

As well as image artefacts, vessel motion can also affect the quantification of calcium scores owing to the partial volume effect.^14–15^ Patients in this study were not prescribed rate controlling medications before their appointment, but this may be considered in the future to help reduce issues with the heart rate.

## Learning points

1. It should not be assumend that manufacturer-recommended exposure settings are optimal.
2. A phantom can be used to investigate characteristic noise variations between scanners.
3. Motion due to patient breathing, arrhythmias and heart rate significantly impact on image quality. Scanner hardware limitations will affect the likelihood of these.

## Consent

None required.
